# Apparent total tract nutrient digestibility of frozen raw, freeze-dried raw, fresh, and extruded dog foods and their effects on serum metabolites and fecal characteristics, metabolites, and microbiota of healthy adult dogs

**DOI:** 10.1093/tas/txae163

**Published:** 2024-11-26

**Authors:** Elizabeth L Geary, Patrícia M Oba, James R Templeman, Kelly S Swanson

**Affiliations:** Department of Animal Sciences, University of Illinois Urbana-Champaign, Urbana, IL, 61801, USA; Department of Animal Sciences, University of Illinois Urbana-Champaign, Urbana, IL, 61801, USA; Primal Pet Foods, Primal Pet Group, Fairfield, CA, 94534USA; Department of Animal Sciences, University of Illinois Urbana-Champaign, Urbana, IL, 61801, USA; Division of Nutritional Sciences, University of Illinois Urbana-Champaign, Urbana, IL, 61801, USA; Department of Veterinary Clinical Medicine, University of Illinois Urbana-Champaign, Urbana, IL, 61801, USA

**Keywords:** canine nutrition, diet format, pet food

## Abstract

Various pet food diet formats are available, but many are poorly studied. The objective of this study was to determine the apparent total tract macronutrient digestibility (ATTD) of frozen raw, freeze-dried raw, fresh, and extruded dog foods and assess their effects on serum metabolites, hematology, and fecal characteristics, metabolites, and microbiota of healthy adult dogs. Ten beagle dogs (4.10 ± 0.74 yr) were used in a replicated 5 × 5 Latin square study to test the following diets: Chicken and Barley Recipe (extruded; Hill’s Science Diet [EXT]), Chicken and White Rice Recipe (fresh; Just Food for Dogs [FRSH]), Chicken Formula (frozen raw; Primal [FRZN]), Chicken and Sorghum Hybrid Freeze-dried Formula (freeze-dried raw; Primal [HFD]), and Chicken Dinner Patties (freeze-dried raw; Stella & Chewy’s [FD]). The experiment was composed of five 35-d periods, with each ending with fecal and blood collections. Data were analyzed using Mixed Models in SAS 9.4, with *P* < 0.05 being significant. Treatment was a fixed effect and dog a random effect. Protein ATTD was higher for FRZN and FD than other diets and higher for HFD than FRSH and EXT. Fat ATTD was higher for HFD than FRZN and EXT and lower for EXT than other diets. Fecal output was higher for dogs fed EXT than those fed other diets and higher for dogs fed FRSH than those fed FRZN, HFD, or FD. Fecal pH was lower in dogs fed EXT and FRSH than those fed other diets. Fecal scores were higher (looser) in dogs fed EXT and FRSH than those fed FRZN and FD. Fecal dry matter was higher in dogs fed FD than those fed other diets and higher in those fed FRZN and HFD than those fed EXT and FRSH. In general, fecal short-chain fatty acids were highest in dogs fed EXT, intermediate in dogs fed FRSH and HFD, and lowest in dogs fed FRZN and FD. Fecal isobutyrate and isovalerate were highest in dogs fed HFD, lowest in dogs fed FRSH, and intermediate in dogs fed other diets. Fecal primary bile acids were higher, while secondary bile acids were lower in dogs fed FRSH than in dogs fed other diets. Fecal microbiota were greatly impacted by diet, with alpha diversity, beta diversity, and relative abundances of over 40 bacterial genera being different among treatments. This study shows that dietary format may lead to great differences in nutrient digestibility and fecal characteristics, metabolites, and microbiota. More research is needed to distinguish the effects of ingredient source, processing method, and nutrient composition.

## Teaser

This study aimed to increase the knowledge base of dog foods differing in processing type. It determined the apparent total tract macronutrient digestibility of frozen raw, freeze-dried raw, fresh, and extruded dog foods and to assess their effects on serum metabolite concentrations, hematology, and fecal characteristics, metabolites, and microbiota of healthy adult dogs.

## Introduction

Alternatives to extruded and retorted dog food are becoming more prevalent, with various processing formats now commercially available. Raw dog food is one format that is growing in popularity, with a growth of 14.14% from 2021 to 2022 and comprising 3.91% of the U.S. market share in 2022 (Atlas Stackline, 2022; Nielsen IQ Byzzer, 2023). Raw dog foods are generally processed in frozen and freeze-dried formats, with these segments comprising 1.65% and 1.43% of the market share in 2022, having experienced 19.10% and 14.50% growth from 2021 to 2022, respectively (Atlas Stackline, 2022; Nielsen IQ Byzzer, 2023). Extruded and retorted dog foods remain the largest segments of the market, but the rapid growth of these emerging forms justifies their research.

How dog owners perceive their pets has shifted over the years, with 85% of owners currently considering their dogs as family members (AVMA, 2018) whereas historically most dogs were viewed as a companion or owned for their work. The dog’s status in the household influences what dog food the owners purchase, with “family member” owners more likely to prioritize health-promoting dog foods (Tesform and Birch, 2010). However, a growing mistrust of commercial diets and pet food manufacturing is causing some pet owners to turn away from traditional foods, as this group becomes increasingly concerned about the quality, safety, and nutritional value of conventional commercial pet food formats (Michel et al., 2008; Morgan et al., 2017; Empert-Gallegos et al., 2020). Many factors may shape pet owners’ rationale for feeding an alternative diet, including cultural or ideological motivations. As well, some owners believe that dogs should eat a diet similar to their own (e.g., human-grade, organic, vegan, fresh; Schlesinger and Joffe, 2011), while others want to feed their dogs similarly to what they perceive domesticated dogs’ ancestors ate (e.g., raw, high-protein, grain-free; Morgan et al., 2017, 2022). Owners may also believe certain diet constituents (e.g., corn, wheat, grains, carbohydrates, additives, and byproducts) are inappropriate for dogs to consume (Michel, 2006; Michel et al., 2008; Morelli et al., 2019). Raw or minimally processed pet foods might be favored because of the notion that high heat, such as would be applied during extrusion or retort, may cause the destruction of critical nutrients (Michel et al., 2008). Furthermore, many people use food to bond with their pets and want to maximize their eating experience with highly palatable foods and diet variety. For the multitude of reasons discussed above, some pet owners want to feed their dogs an alternative diet, such as home-prepared, organic, human-grade, mildly cooked/fresh, vegetarian/vegan, or raw diets.

Raw dog food has historically been and continues to be, a controversial subject. With the conflicting purported risks and benefits of raw feeding (Beloshapka et al., 2012; Freeman et al., 2013; Algya et al., 2018; Vecchiato et al., 2022), it is imperative to scrutinize the evidence and fill the research gaps. One of the major arguments for why dogs should consume raw food high in animal protein is because the diet profile is similar to the wolves’ diet (Bosch et al., 2014). However, considering the vast environmental, biological, and lifestyle differences between wolves and dogs, the diet of wolves cannot be extrapolated with certainty as being beneficial for dogs (Axelsson et al., 2013; Bosch et al., 2014; Remonti et al., 2015). Similarly, proponents of raw diets often argue that dogs should consume more meat products (Prata, 2022), mentioning that some plant proteins are less digestible, have poor amino acid profiles, or contain antinutritional factors. On the other hand, opponents of raw feeding argue that even commercial raw diets might be at greater risk for a nutrient imbalance (Freeman et al., 2013; Vecchiato et al., 2022). However, as is the case for all types of pet food, raw diets pose no nutrient imbalance risk if properly formulated. Another argument against raw diets is an increased risk of bacterial contamination due to the lack of a heat-induced kill step (Davies et al., 2019). The amount of *Salmonella* contamination reported in commercial raw diets varies, with most studies reporting around 20% contamination of diets tested (Weese et al., 2005; Vecchiatio et al., 2022). However, as per the FDA, there is a zero-tolerance policy for pathogen contamination in all commercial pet foods. Additionally, extruded pet food and other types of pet food manufacturing processes can also have bacterial contamination (FDA, 2023). To add, there are technologies that exist and are widely applied across the industry to ensure the safety of pet foods. For example, high-pressure processing prevents bacterial growth by causing water molecules to change their bond configuration, thereby reducing water activity (San Martín et al., 2002). Moreover, freeze-drying of raw foods also reduces water activity to prevent bacterial growth (Shukla, 2011).

Due to the purported benefits of feeding less-processed foods, mildly cooked or minimally processed foods are also gaining popularity. Mildly cooked foods are broadly characterized as pet foods that have been heat treated without excessive heat damage. While high heat (above 100 °C) can cause a decrease in the digestibility of proteins due to protein aggregation, oxidation, cross-linking, and increased disulfide bridges, low-temperature heating can increase digestibility due to partial protein unfolding and exposing cleavage sites (Bhat et al., 2021). Mildly cooked dog foods are processed at relatively low temperatures (75 to 95 °C) to kill pathogens without causing a detrimental loss in digestibility (Kerr et al., 2012; Algya et al., 2018; Oba et al., 2019; Do et al., 2021; Roberts et al., 2023). Extrusion, however, requires greater processing temperatures (125 to 150 °C; Rokey et al., 2010), so a reduction in digestibility may result.

Despite their increasing popularity, there is a paucity of research on these emerging diet formats. Therefore, a primary objective of this study was to determine the apparent total tract macronutrient digestibility (ATTD) of extruded, fresh, frozen raw, and freeze-dried raw dog diets. A secondary objective of the study was to determine the serum metabolite concentrations, hematology, fecal characteristics, metabolites, and microbiota of healthy adult dogs consuming extruded, fresh, frozen raw, and freeze-dried raw dog diets. We hypothesized that the raw and fresh diets would have a greater ATTD than the extruded diet. Additionally, we hypothesized that fecal scores would not differ among dogs fed different diets, but that fecal dry matter (DM) percentage would be lower in dogs fed the raw or fresh diets than those fed the extruded diet. Finally hypothesized that dogs fed the extruded diet would have higher short-chain fatty acid (SCFA) and dogs fed the raw diets would have higher fecal branched-chain fatty acid (BCFA) concentrations.

## Materials and Methods

All animal care procedures were approved by the University of Illinois Institutional Animal Care and Use Committee prior to the initiation of the experiment (IACUC #22010).

### Animals and Housing

Ten healthy spayed female adult beagles [body weight (BW) = 8.60 ± 0.83 kg; age = 4.10 ± 0.74 yr old; body condition score (BCS) = 5.35 ± 0.47] were used in a replicated 5 × 5 Latin square design. Dogs were housed in an environmentally (temperature and light) controlled room in an animal facility at the University of Illinois Urbana-Champaign. Dogs were housed individually in runs (approximately 1.2 m wide × 2.4 m long) for the duration of the study. The room was on a 14 h light: 10 h dark schedule, with lights being on from 7:00 to 21:00. Dogs had free access to fresh water at all times. Dogs were fed twice a day (8:00 and 17:00). The amount of food offered was initially based on previous feeding records and the estimated caloric content of the diets given by the manufacturers, but then adjusted weekly to maintain BW. Daily food intake was recorded. Dogs had access to toys at all times and were socialized at least two times per week, where they were given other toys, further enrichment, and socialization with each other and humans.

### Experimental Timeline and Diets

The study consisted of five 35-d experimental periods, with each consisting of a diet adaption phase (7 d), treatment phase (21 d), and collection phase (7 d). Dogs were adapted to the new dietary treatment at the beginning of each experimental period (days 1 to 2: 75% kcal from prior dietary treatment + 25% kcal from new dietary treatment; days 3 to 4: 50% kcal from prior dietary treatment + 50% kcal from new dietary treatment; days 5 to 6: 25% kcal from prior dietary treatment + 75% kcal from new dietary treatment; day 7: 100% kcal from new dietary treatment).

Test diets included: an extruded kibble diet (Hill’s Science Diet Adult Chicken & Barley Recipe; Hill’s Pet Nutrition, Topeka, KS [EXT]), a mildly cooked human-grade fresh diet (Chicken & White Rice Recipe; Just Food For Dogs, Irvine, CA [FRSH]), a frozen raw diet (Primal Frozen Nuggets Chicken Formula; Primal Pet Foods, Fairfield, CA [FRZN]), a hybrid freeze-dried raw diet (Primal Hybrid Freeze-Dried Chicken & Sorghum Formula; Primal Pet Foods [HFD]), and a freeze-dried raw diet (Chicken Dinner Patties; Stella & Chewy’s, Milwaukee, WI [FD]; **Table 1**). All dietary treatments were commercial diets formulated to meet all Association of American Feed Control Officials (AAFCO, 2023) nutrient recommendations for the maintenance of adult dogs. At the end of each treatment phase, there was a collection phase comprised of a 5-d total fecal collection, with the first day serving for fresh fecal collection, and 2 d for blood collection. Dogs were weighed and BCS were assessed using a 9-point scale (Laflamme, 1997) once a week prior to the morning feeding.

**Table 1.** Analyzed chemical composition and ingredients of dog diets tested

Item	EXT^*^	FRSH^†^	FRZN^‡^	HFD^‖^	FD^$^
Dry matter, DM, %	92.89	30.82	22.69	95.43	94.76
------ DM basis ------					
Protein, %	24.61	31.06	46.70	35.17	56.75
Acid-hydrolyzed fat, %	15.41	15.30	33.83	30.51	30.71
Total dietary fiber, %	9.58	2.21	3.48	3.88	1.90
Ash, %	5.21	6.51	7.40	9.23	10.13
Nitrogen-free extract, %	45.19	44.92	8.59	21.21	0.51
Total starch, %	45.64	44.80	0.00	15.17	0.00
Gelatinized starch, %	35.21	40.06	0.00	12.86	0.00
Cook, %	89.40	89.40	—	84.70	—
Gross energy, kcal/g	4.97	5.01	6.40	5.76	6.01
Metabolizable energy^¶^, kcal/g	4.18	4.42	5.26	5.00	5.05
Metabolizable energy^**^, kcal/g	3.75	3.96	4.81	4.57	4.61

^*^Extruded diet (EXT); Chicken and Barley Recipe (Hill’s Pet Nutrition, Topeka, KS); ingredients: chicken, whole grain wheat, cracked pearled barley, whole grain sorghum, whole grain corn, corn gluten meal, chicken meal, chicken fat, chicken liver flavor, dried beet pulp, soybean oil, pork flavor, lactic acid, flaxseed, potassium chloride, choline chloride, iodized salt, calcium carbonate, vitamins (vitamin E supplement, l-ascorbyl-2-polyphosphate (source of vitamin C), niacin supplement, thiamin mononitrate, vitamin A supplement, calcium pantothenate, riboflavin supplement, biotin, vitamin B12 supplement, pyridoxine hydrochloride, folic acid, vitamin D3 supplement), minerals (ferrous sulfate, zinc oxide, copper sulfate, manganous oxide, calcium iodate, sodium selenite), taurine, oat fiber, mixed tocopherols for freshness, natural flavors, beta-carotene, apples, broccoli, carrots, cranberries, green peas.

^†^Fresh diet (FRSH); Chicken and White Rice Recipe (Just Food for Dogs, Irvine, CA); ingredients: chicken thigh, long grain white rice, spinach, carrots, apples, chicken gizzard, chicken liver, eicosapentaenoic acid (EPA) and docosahexaenoic acid (DHA), dicalcium phosphate dihydrate, calcium, sodium chloride, choline bitartrate, dried seaweed meal, zinc oxide, magnesium amino acid chelate, vitamin E (as a-tocopherol succinate), ferrous amino acid chelate, copper amino acid chelate, vitamin D3 (as cholecalciferol), vitamin B5 (as calcium d-pantothenate), riboflavin, vitamin B12 (as cyanocobalamin).

^‡^Frozen raw diet (FRZN); Chicken Formula (Primal Pet Group, Fairfield, CA); ingredients: chicken (with ground bone), chicken livers, organic carrots, organic squash, organic kale, organic apples, organic pumpkin seeds, organic sunflower seeds, organic broccoli, organic blueberries, organic cranberries, organic parsley, organic apple cider vinegar, montmorillonite clay, fish oil, organic quinoa, organic coconut oil, vitamin E supplement, organic ground alfalfa, dried organic kelp, zinc sulfate, liquid *Lactobacillus acidophilus* fermentation product, liquid *Lactobacillus casei* fermentation product, liquid *Lactobacillus reuteri* fermentation product, liquid *Bifidobacterium animalis* fermentation product.

^‖^Hybrid freeze-dried raw diet (HFD); Chicken and Sorghum Hybrid Freeze-Dried Formula (Primal Pet Group, Fairfield, CA); ingredients: chicken, sorghum, chicken livers, chicken fat, inulin, apple pomace, dicalcium phosphate, potassium chloride, salt, salmon oil, choline chloride, vitamin and mineral premix (sodium chloride, dl-alpha-tocopheryl acetate, ainc sulfate, ferrous sulfate, biotin, retinol palmitate, manganese sulfate, niacin, d-calcium pantothenate, cholecalciferol, sodium selenite, copper sulfate anhydrous, thiamin mononitrate, riboflavin, cyanocobalamin, pyridoxine hydrochloride, potassium iodide, folic acid), vegetable oil, liquid *Lactobacillus acidophilus* fermentation product, liquid *Lactobacillus casei* fermentation product, liquid *Lactobacillus reuteri* fermentation product, liquid *Bifidobacterium animalis* fermentation product, rosemary extract.

^$^Freeze-dried raw diet (FD); Chicken Dinner Patties (Stella & Chewy’s, Milwaukee, WI); ingredients: chicken with ground bone, chicken liver, chicken gizzard, pumpkin seed, organic cranberries, organic spinach, organic broccoli, organic beets, organic carrots, organic squash, organic blueberries, fenugreek seed, potassium chloride, dried kelp, sodium phosphate, tocopherols (preservative), choline chloride, dried *Pediococcus acidilactici* fermentation product, dried *Lactobacillus acidophilus* fermentation product, dried *Bifidobacterium longum* fermentation product, dried *Bacillus coagulans* fermentation product, zinc proteinate, iron proteinate, taurine, calcium carbonate, vitamin E supplement, thiamin mononitrate, copper proteinate, manganese proteinate, sodium selenite, niacin supplement, D-calcium pantothenate, riboflavin supplement, vitamin A supplement, vitamin D3 supplement, vitamin B12 supplement, pyridoxine hydrochloride, folic acid.

^¶^Metabolizable energy estimated using Atwater factors (4 kcal/g for protein and nitrogen-free extract; 9 kcal/g for fat).

^**^Metabolizable energy estimated using modified Atwater factors (3.5 kcal/g for protein and nitrogen-free extract; 8.5 kcal/g for fat).

All five diets had distinct processing methods. The EXT diet was extruded, but the specific parameters were unknown. The FRSH diet was mildly cooked in skillets with a target temperature between 76 and 82 °C for 20 to 40 min and frozen until thawed to feed. The FRZN diet’s raw ingredients were mixed, formed into nugget shapes, and then frozen at −32 °C. It was stored at −20 °C until being thawed for feeding. For the HFD diet, approximately 35% of the included chicken was steamed at 71 °C for 10 min, while the rest remained raw. The sorghum was cooked at a sufficient temperature and time combination to achieve starch gelatinization over 90%. More specifically, sorghum underwent preconditioning at temperatures up to 176 °C for 1 to 3 min, followed by steam exposure at pressures of 120 to 220 psi and temperatures of 260 to 315 °C for 1 to 5 min. The steamed fraction of the chicken and the cooked sorghum were mixed with the raw ingredients, shaped, and frozen at −32 °C for 8 h. The diet was then placed under vacuum while the temperature was raised to −23 °C for 3 h. To ensure the product was thoroughly dried, the temperature was increased slowly to reach an internal temperature of 60 °C over 5 h. The FD was produced by freeze-drying raw ingredients, but the specific conditions were unknown. The processing information for the FRSH, FRZN, and HFD diets were provided by the manufacturers.

### Fecal Scoring, Sample Collection, and Analysis

All fecal samples collected were scored according to the following scale: 1: hard, dry pellets, small hard mass; 2: formed, dry stool, remains firm but soft; 3: soft, formed, and moist stool, retains shape; 4: soft, unformed stool, assumes the shape of container; 5: a watery, liquid that can be poured. Total feces excreted during the collection phase were weighed, frozen at −20 °C, and pooled by the dog prior to analyses and digestibility calculations. Fresh feces (within 15 min of defecation) were collected for measurement of pH, moisture content, bile acid and fermentative metabolite concentrations, and microbiota. Fermentative metabolites of interest included SCFA, which serve as an important energy source for colonocytes, and protein fermentative products (ammonia; BCFA; phenols; indoles) that are responsible for fecal odor and are associated with gastrointestinal disease (Cummings and Macfarlane, 1991).

Fecal pH was measured immediately using an AP10 pH meter (Denver Instrument, Bohemia, NY) equipped with a Beckman Electrode (Beckman Instruments Inc., Fullerton, CA), and then aliquots were collected. Aliquots for phenol and indole analysis were frozen at −20 °C immediately after collection. The aliquot for ammonia, SCFA, and BCFA analyses was collected and placed in 2N hydrochloric acid and frozen at −20 °C. Aliquots for bile acids and microbiota were placed into sterile cryogenic vials (Nalgene, Rochester, NY), quickly frozen in dry ice, and stored at −80 °C until analysis. Fecal DM determination was measured according to AOAC (2006; method 934.01) using a 105 °C oven.

### Fecal Fermentative Metabolite, Bile Acid, Fatty Acid, and Sterol Analysis

Fecal SCFA and BCFA concentrations were determined by gas chromatography according to Erwin et al. (1961) using a gas chromatograph (Hewlett-Packard 5890A series II, Palo Alto, CA) and a glass column (180 cm × 4 mm i.d.) packed with 10% SP-1200/1% H_3_PO_4_ on 80/100 + mesh Chromosorb WAW (Supelco Inc., Bellefonte, PA). Nitrogen was the carrier with a flow rate of 75 mL/min. Oven, detector, and injector temperatures were 125, 175, and 180 °C, respectively. Fecal ammonia concentrations were determined according to the method of Chaney and Marbach (1962). Fecal phenol and indole concentrations were determined using gas chromatography after being extracted with methanol according to the methods described by Flickinger et al. (2003). Fecal bile acids were measured according to Batta et al. (2002) and Blake et al. (2019). Fecal fatty acid and sterol concentrations were analyzed using gas chromatography coupled with mass spectrometry as described by Sung et al. (2023). Briefly, an aliquot of 10 to 14 mg lyophilized stool was added to 160 μL of 1-butanol containing 10 μL of internal standards (d_7_-sitostanol, d_6_-cholesterol, d_4_-stearic acid, and d_4_-cholestane (2 mg/mL each) and 20 µL each of d_4_-cholic acid and d_4_-lithocholic acid (1 mg/mL each) followed by adding 20 μL of hydrochloric acid. Samples were incubated for 4 h at 65 °C. Following incubation, samples were completely evaporated at 65 °C under N gas, 200-μL trimethylsilylation derivatization agent was added and samples were incubated for 30 min. The samples were then evaporated under N gas and resuspended in 200-μL hexane, vortexed, and centrifuged at 5 °C for 10 min at 18.0 × *g*. The supernatant was then analyzed using gas chromatography and mass spectrometry.

### Quantitative Polymerase Chain Reaction Analysis and Dysbiosis Index

Quantitative polymerase chain reaction (qPCR) analysis of select bacterial taxa was performed with specific primers targeting *Faecalibacterium*, *Fusobacterium*, *Blautia*, universal bacteria, *Turicibacter*, *Escherichia coli*, *Clostridium hiranonis*, and *Streptococcus* as described in AlShawaqfeh et al. (2017). Briefly, the conditions for qPCR were as follows: initial denaturation at 98 °C for 2 min, then 40 cycles with denaturation at 98 °C for 3 s, and annealing for 3 s. Melt curve analysis was performed to validate the specific generation of the qPCR product using these conditions: 95 °C for 1 min, 55 °C for 1 min, and increasing incremental steps of 0.5 °C for 80 cycles for 5 s each. Each reaction was run in duplicate. The qPCR data were expressed as the log amount of DNA (fg) for each particular bacterial group/10 ng of isolated total DNA as reported previously (Suchodolski et al., 2012; Panasevich et al., 2015).

The degree of dysbiosis is represented as a single numerical value that measures the closeness of a taxa compared with the mean abundances derived from healthy and diseased populations and is calculated by an Euclidean distance model, as detailed in AlShawaqfeh et al. (2017). The dysbiosis index quantifies the degree of gut dysbiosis, increasing with the amplified clinical severity of the disease (Galler et al., 2022). Using this system, a score less than zero is considered healthy and “normal,” whereas a score of two or higher denotes extreme dysbiosis. A dysbiosis index ranging from zero to two is an equivocal outcome. However, any sample with values outside the reference range for each bacteria quantified may denote a state of mild dysbiosis (Galler et al., 2022).

### *Fecal Microbiota* Analysis

Fecal bacterial DNA was extracted according to the manufacturer’s instructions using the MO BIO PowerSoil Kit (MO BIO Laboratories, Carlsbad, CA) with bead beating using a vortex adaptor, followed by quantification of extracted DNA using a Qubit® 3.0 Fluorometer (Life Technologies, Grand Island, NY). DNA quality was determined using an E-Gel Power Snap Electrophoresis Device (Invitrogen, Waltham, MA) on E-Gel EX 1% Agarose Gels. 16S rRNA gene amplicons were generated using a Fluidigm Access Array (Fluidigm Corporation, South San Francisco, CA) in combination with Roche High Fidelity Fast Start Kit (Roche, Indianapolis, IN). The primers 515F (5ʹGTGCCAGCMGCCGCGGTAA-3ʹ) and 806R (5ʹ-GGACTACHVGGGTWTCTAAT-3ʹ) that target a 252 bp-fragment of the V4 region of the 16S rRNA gene were used for amplification (primers synthesized by IDT Corp., Coralville, IA) (Caporaso et al., 2012). The CS1 forward and CS2 reverse tags were added according to the Fluidigm protocol. The quality of the amplicons were assessed using a Fragment Analyzer (Advanced Analytics, Ames, IA) to confirm amplicon region and size. A DNA pool was generated by combining equimolar amounts of the amplicons from each sample. The pooled samples were then size-selected on a 2% agarose E-gel (Life technologies, Grand Island, NY) and extracted using a Qiagen gel purification kit (Qiagen, Valencia, CA). Cleaned size-selected pooled products were run on an Agilent Bioanalyzer to confirm the appropriate profile and average size. Illumina sequencing was performed on a MiSeq using v3 reagents (Illumina Inc., San Diego, CA) at the Roy J. Carver Biotechnology Center at the University of Illinois. Illumina 16S rRNA gene amplicon sequencing produced a total of 2,032,342 sequences, with an average of 40,647 sequences per sample. Forward reads were trimmed using the FASTX Toolkit (version 0.0.14), and the resulting sequence data were analyzed using QIIME 2.0, version 2022.8.3 (Caporaso et al., 2010). Raw sequence amplicons were imported into the QIIME2 package and analyzed by the DADA2 pipeline for quality control (QC value ≥20; Callahan et al., 2016). Samples were then rarefied to 11,526 reads and used for analysis. Subsequent samples were assigned to taxonomic groups with the SILVA database (SILVA 138 99% ASV from 515F/806R region of sequences, with the QIIME2 classifier trained on 515F/806R V4 region of 16S) (Bokulich et al., 2018; Robeson et al., 2021). The rarefied samples were used for analysis of alpha and beta diversity. Principal coordinate analysis was performed using weighted and unweighted unique fraction metric (UniFrac) distances (Lozupone and Knight, 2005).

### Blood Sample Collection and Analysis

At the end of each period, overnight fasted blood was collected via jugular puncture. Approximately 9 mL of blood was collected for serum metabolite concentrations, hematology, and canine pancreatic lipase immunoreactivity (cPLI). Samples were immediately transferred to appropriate vacutainer tubes, with 0.5 mL going into #367841 BD Vacutainer Plus plastic whole blood tubes (Lavender with K_2_EDTA additive) and 8.5 mL going into #367974 BD Vacutainer Plus plastic serum tubes (red/gray with clot activator and gel for serum separation; BD, Franklin Lakes, NJ). Blood tubes for serum isolation were centrifuged at 1,300 × *g* at 4 °C for 10 min (Beckman CS-6R centrifuge; Beckman Coulter Inc., Brea, CA) and then aliquoted into cryovials. One cryovial of serum was transported to the University of Illinois Veterinary Medicine Diagnostics Laboratory for serum chemistry analysis using a Hitachi 911 clinical chemistry analyzer (Roche Diagnostics, Indianapolis, IN). An additional aliquot of serum was sent to Texas A&M University for cPLI analysis using an immunoassay. K_2_EDTA tubes were cooled (but not frozen) and transported to the University of Illinois Veterinary Medicine Diagnostics Laboratory for hematology.

### *Chemical* Analyses

Wet diets (FRSH and FRZN) were first freeze-dried. Fecal samples were dried at 55 °C in a forced-air oven. All diets and fecal samples were then ground in a Wiley mill (model 4, Thomas Scientific, Swedesboro, NJ) through a 2-mm screen. Diets and fecal samples were analyzed for DM and ash according to AOAC (2006; methods 934.01 and 942.05), with organic matter (OM) calculated. Crude protein (CP) was calculated from Leco (FP2000 and TruMac) total nitrogen values according to AOAC (2006; method 992.15). Total lipid content (acid-hydrolyzed fat) was determined according to the methods of the American Association of Cereal Chemists (1983; method 30-14.01) and Budde (1952). Gross energy was measured using an oxygen bomb calorimeter (model 6200, Parr Instruments, Moline, IL). The total dietary fiber concentrations of diets were determined according to Prosky et al. (1992) by Eurofins (Eurofins Nutrition Analysis Center, Des Moines, IA).

### Statistical Analyses

Data were analyzed using the Mixed Models (MIXED) procedure of SAS 9.4 (SAS Institute, Inc., Cary, NC). The fixed effect of treatment was tested, and the dog was considered a random effect. Data were tested for normality using the UNIVARIATE procedure of SAS. Differences between treatments were determined using a Fisher-protected least significant difference with a Tukey adjustment to control for experiment-wise error. A *P* < 0.05 was accepted as statistically significant. Reported pooled standard errors of the mean were determined according to the Mixed Models procedure of SAS.

## Results

### Food Intake, BW, and BCS

Besides cyclical bouts of diarrhea from most dogs consuming the FD diet, all dogs remained healthy throughout the experiment. As-is food intake (g/d) differed according to the moisture content of the diets, with dogs fed high-moisture diets consuming more (*P* < 0.0001) food than those fed dry diets (**Table 2**). On a DM basis, however, the food intake of dogs fed the EXT diet was higher (*P* < 0.0001) than that of dogs fed the other diet types. Nutrient and caloric intakes differed greatly across dietary treatments due to the variance in dietary nutrient and energy concentrations. Even though food intakes were adjusted to maintain BW and BCS throughout the entire study, differences in BW were noted.

**Table 2.** Apparent total tract macronutrient and energy digestibility and body weight, body condition score, food intake, and fecal output of dogs fed extruded, fresh, and raw foods

Item	EXT^*^	FRSH	FRZN	HFD	FD	SEM^†^	*P*-value
Body weight (kg)	8.64^bc^	8.44^c^	8.70^ab^	8.85^a^	8.86^a^	0.2392	<0.0001
Body condition score^‡^	5.40	5.35	5.35	5.44	5.41	0.1263	0.9831
Food intake							
g food/d (as-is)	144.52^c^	320.51^b^	417.05^a^	119.05^d^	114.84^d^	14.05	<0.0001
g dry matter/d	134.24^a^	98.78^cd^	94.63^d^	113.61^b^	108.82^bc^	5.047	<0.0001
g organic matter/d	127.25^a^	92.35^bc^	87.62^c^	103.12^b^	97.80^bc^	4.693	<0.0001
g crude protein/d	33.04^c^	30.68^c^	44.19^b^	39.96^b^	61.75^a^	1.881	<0.0001
g fat/d	20.69^b^	15.11^c^	32.01^a^	34.66^a^	33.42^a^	1.328	<0.0001
Modified Atwater kcal/d^‖^	503.40^ab^	391.17^c^	455.16^b^	519.20^a^	501.65^ab^	21.84	<0.0001
Atwater kcal/d^$^	561.12^ab^	436.61^c^	497.75^bc^	568.05^a^	549.53^ab^	24.05	<0.0001
TME_n_ kcal/d^¶^	571.86^ab^	456.36^c^	554.52^ab^	613.49^a^	540.83^b^	25.72	<0.0001
Fecal output							
Fecal output, as-is (g/d)	90.22^a^	55.51^b^	37.33^c^	38.49^c^	39.80^c^	3.926	<0.0001
Fecal output, dry matter (g/d)	24.96^a^	14.27^b^	14.09^b^	13.26^b^	15.45^b^	1.086	<0.0001
As-is fecal output (g/d)/dry matter intake (g/d)	0.67^a^	0.56^b^	0.40^c^	0.34^c^	0.37^c^	0.0228	<0.0001
Digestibility, %							
Dry matter	81.53^c^	85.62^b^	85.06^b^	88.24^a^	85.69^b^	0.5710	<0.0001
Organic matter	83.93^c^	89.83^b^	90.33^b^	91.21^ab^	92.39^a^	0.4436	<0.0001
Crude protein	81.99^d^	85.91^c^	93.00^a^	89.83^b^	94.17^a^	0.6666	<0.0001
Fat	92.14^c^	95.19^b^	94.28^b^	96.84^a^	95.75^ab^	0.4149	<0.0001
Energy	83.76^d^	89.87^c^	91.41^bc^	91.96^ab^	93.21^a^	0.4518	<0.0001

^*^EXT: Chicken and Barley Recipe (Hill’s Pet Nutrition, Topeka, KS); FRSH: Chicken and White Rice Recipe (Just Food for Dogs, Irvine, CA); FRZN: Chicken Formula (Primal Pet Group, Fairfield, CA); HFD: Chicken and Sorghum Hybrid Freeze-Dried Formula (Primal Pet Group, Fairfield, CA); FD: Chicken Dinner Patties (Stella & Chewy’s, Milwaukee, WI).

^†^Pooled standard error of the mean.

^‡^9-point scale (Laflamme, 1997).

^‖^Calculated using modified Atwater values (3.5 kcal/g for protein and nitrogen-free extract; 8.5 kcal/g for fat).

^$^Calculated using Atwater values (4 kcal/g for protein and nitrogen-free extract; 9 kcal/g for fat).

^¶^Calculated using TME_n_ values (Geary et al., 2023).

^a-d^Means lacking a common superscript differ (*P* < 0.05).

### *Fecal Output and Apparent Total Tract Macronutrient* Digestibility

Dogs fed the EXT diet had the highest (*P* < 0.0001) fecal output on a wet (as-is) and DM basis (**Table 2**). Dogs fed the FRSH diet had higher (*P* < 0.0001) wet fecal output than dogs fed the FRZN, HFD, and FD diets. The fecal output/DM intake ratio data followed the same pattern and statistical differences. The EXT diet had lower (*P* < 0.0001) DM, OM, CP, fat, and energy ATTD than all the other diets tested. The DM ATTD was lower (*P* < 0.0001) for the FRSH, FRZN, and FD diets than for the HFD diet. The OM ATTD was lower (*P* < 0.0001) for the FRSH and FRZN diets than the FD diet. The CP ATTD was lower (*P* < 0.0001) for the FRSH diet than the FRZN, FD, and HFD diets, and lower (*P* < 0.0001) for the HFD diet than the FRZN and FD diets. The fat ATTD was also lower (*P* < 0.0001) for the FRSH and FRZN diets than the HFD diet. The energy ATTD was lower (*P* < 0.0001) for the FRSH diet than the HFD and FD diets, and lower (*P* < 0.0001) for the FD diet than the FRZN diet.

### *Fecal* Characteristics

**Figure 1.**
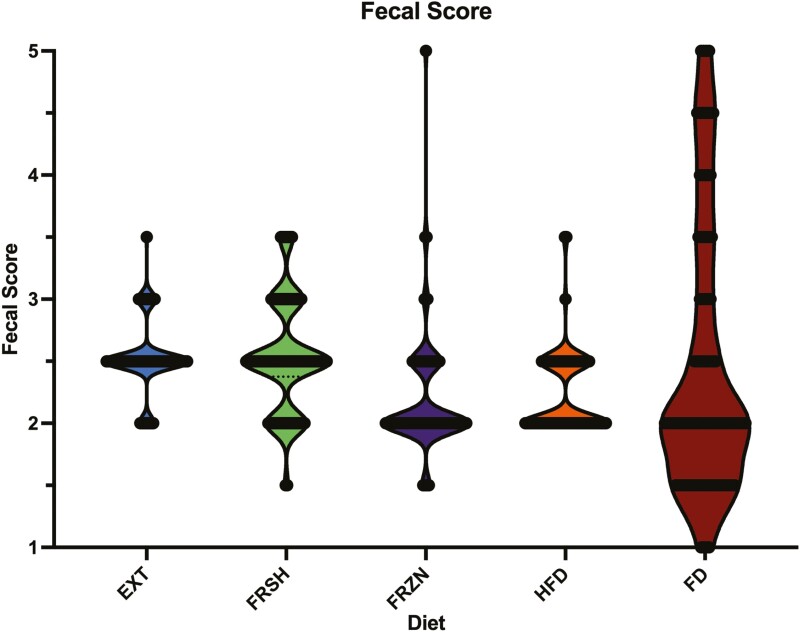
Violin plot of all fecal scores of dogs fed extruded, fresh, and raw foods.

Although all average fecal scores were within the normal range (2 to 3 out of 5), dogs fed the EXT and FRSH diets had higher (looser; *P* = 0.0186) fecal scores than those fed the FRZN and FD diets, with those fed the HFD diet being intermediate (**Table 3**). However, dogs fed the FD diet had a greater variation than the other diets, with several fecal scores being outside the normal range (**Figure 1**). Fecal pH was higher (*P* < 0.0001) in dogs fed the FRZN, HFD, and FD diets than those fed the EXT and FRSH diets. Dogs fed the FD diet had a higher (*P* < 0.0001) fecal DM % than those fed the EXT and FRSH diets. Also, dogs fed the FRZN diet had a higher (*P* < 0.0001) fecal DM % than those fed the FRSH diet.

**Table 3.** Fecal characteristics and metabolite concentrations of dogs fed extruded, fresh, and raw foods

Item	EXT^*^	FRSH	FRZN	HFD	FD	SEM^†^	*P*-value
Fecal characteristics							
Fecal score^‡^	2.75^a^	2.75^a^	2.20^b^	2.40^ab^	2.25^b^	0.2297	0.0186
Fecal pH	5.52^b^	5.16^b^	6.52^a^	6.62^a^	6.97^a^	0.1225	<0.0001
Fecal DM%	27.62^bc^	25.56^c^	35.90^ab^	33.58^abc^	41.17^a^	2.372	<0.0001
Fecal metabolites, µmol/g DM							
Acetate	441.40^a^	232.80^bc^	233.63^bc^	252.09^b^	193.17^c^	26.27	<0.0001
Propionate	192.16^a^	94.66^c^	99.44^c^	149.07^b^	77.56^c^	10.99	<0.0001
Butyrate	170.37^b^	256.09^a^	51.99^c^	101.70^bc^	47.20^c^	21.82	<0.0001
Total SCFA^‖^	803.92^a^	583.55^ab^	385.06^cd^	502.86^bc^	317.92^d^	45.78	<0.0001
SCFA as a % of total							
Acetate, %	55.23^ab^	41.92^c^	60.89^a^	50.32^b^	59.25^a^	2.138	<0.0001
Propionate, %	24.17^b^	16.45^c^	25.80^ab^	29.87^a^	26.09^ab^	1.180	<0.0001
Butyrate, %	20.60^y^	41.63^z^	13.31^y^	19.81^y^	14.66^y^	2.375	0.0003
Isobutyrate	7.83^b^	2.67^c^	7.93^b^	14.34^a^	8.54^b^	0.9717	<0.0001
Isovalerate	9.73^cd^	6.06^d^	11.89^bc^	21.99^a^	15.97^b^	1.484	<0.0001
Valerate	4.63^ab^	5.53^a^	1.66^c^	4.95^ab^	2.19^bc^	0.7832	0.0008
Total BCFA^‖^	22.19^bc^	14.26^c^	21.48^bc^	41.28^a^	26.70^b^	2.554	<0.0001
Phenol	0.49^b^	0.45^b^	1.35^a^	0.82^b^	1.30^a^	0.1519	<0.0001
4-methylphenol	0.69^ab^	0.77^a^	0.45^b^	0.73^ab^	0.49^ab^	0.0864	0.0107
4-ethylphenol	2.20^b^	0.75^c^	2.27^ab^	3.26^a^	2.61^ab^	0.2814	<0.0001
Indole	0.92^b^	0.20^c^	2.42^a^	4.16^a^	2.45^a^	0.2247	<0.0001
2,3-dimethylindole	0.12	0.12	0.13	0.10	0.07	0.0201	0.1322
Total P/I^‖^	4.58^c^	2.33^d^	6.62^bc^	9.09^a^	6.91^b^	0.5550	<0.0001
Ammonia	115.09^yz^	66.36^y^	70.41^y^	122.72^z^	121.84^yz^	25.00	0.0013

^*^EXT: Chicken and Barley Recipe (Hill’s Pet Nutrition, Topeka, KS); FRSH: Chicken and White Rice Recipe (Just Food for Dogs, Irvine, CA); FRZN: Chicken Formula (Primal Pet Group, Fairfield, CA); HFD: Chicken and Sorghum Hybrid Freeze-Dried Formula (Primal Pet Group, Fairfield, CA); FD: Chicken Dinner Patties (Stella & Chewy’s, Milwaukee, WI).

^†^Pooled standard error of the mean.

^‡^Fecal score: 1 = hard, dry pellets, small hard mass; 2 = hard formed, dry stool, remains firm and soft; 3 = soft, formed and moist stool, retains shape; 4 = soft, unformed stool, assumes shape of container; 5 = watery, liquid that can be poured.

^‖^Total SCFA = acetate + propionate + butyrate; total BCFA = valerate + isovalerate + isobutyrate; total P/I = phenol + 4-methylphenol + 4-ethylphenol + indole + 7-methylindole + 3-methylindole + 2,3-dimethylindole.

^a-d^Means lacking a common superscript differ (*P* < 0.05).

^y-z^Means lacking a common superscript differ using Wilcoxon (*P* < 0.05).

### Fecal Fermentative Metabolite, Bile Acid, Fatty Acid, and Sterol Concentrations

Dogs fed the EXT diet had higher (*P* < 0.0001) fecal total SCFA concentrations than dogs fed the FRZN, HFD, and FD diets (**Table 3**). Also, dogs fed the FRSH diet had higher (*P* < 0.0001) fecal total SCFA concentrations than dogs fed the FRZN and FD diets. Lastly, dogs fed the HFD diet had higher (*P* < 0.0001) fecal total SCFA concentrations than dogs fed the FD diet. Fecal acetate and propionate concentrations followed a similar pattern, with dogs fed the EXT diet having the highest (*P* < 0.0001) concentrations, dogs fed the FD diet having the lowest concentrations, and dogs fed the other diets being intermediate. Concentrations of fecal butyrate had a slightly different pattern, as dogs fed the FRSH diet had higher (*P* < 0.0001) fecal butyrate concentrations than dogs fed all other diets. Dogs fed the EXT diet had higher (*P* < 0.0001) fecal butyrate concentrations than dogs fed the FRZN and FD diets. In terms of molar ratios (% of total), acetate was highest (*P* < 0.0001) in dogs fed FRZN and FD, propionate was highest (*P* < 0.0001) in dogs fed HFD, and butyrate was highest (*P* = 0.0003) in dogs fed FRSH.

Dogs fed the HFD diet had higher (*P* < 0.0001) fecal total BCFA and total phenol + indole concentrations than dogs fed all other diets. Also, dogs fed the FD diet had higher (*P* < 0.0001) fecal total BCFA concentrations than dogs fed the FRSH diet. Dogs fed the FD diet also had higher (*P* < 0.0001) fecal total phenol + indole concentrations than dogs fed the EXT and FRSH diets. Most individual BCFA, phenols, and indoles followed similar patterns. Fecal ammonia concentrations were higher (*P* = 0.0013) in dogs fed the HFD diet than those fed the FRSH and FRZN diets.

Fecal bile acid concentrations varied widely among diets (**Table 4**). Dogs fed the FRZN diet had higher (*P* < 0.0001) fecal total bile acid concentrations than those fed EXT, FRSH, and HFD. Also, dogs fed the HFD and FD diets had higher (*P* < 0.0001) fecal total bile acid concentrations than dogs fed the EXT diet. Fecal secondary bile acid concentrations followed a similar pattern. Fecal primary bile acid concentrations were different, however. Dogs fed the FRSH diet had higher (*P* < 0.0001) fecal total primary bile acid concentrations than dogs fed all other diets, with individual primary bile acids following a similar pattern. These differences in primary and secondary bile acid concentrations led to differences in primary and secondary bile acid percentages. Dogs fed the FRSH diet had higher (*P* < 0.0001) fecal primary bile acid percentage and lower (*P* < 0.0001) fecal secondary bile acid percentage than dogs fed all other diets, leading to much higher primary:secondary bile acid ratio for dogs fed that treatment. Dogs fed the EXT, FRZN, or HFD diets had greater (*P* < 0.0001) fecal bai gene abundance than dogs fed the FRSH or FD diets.

**Table 4.** Fecal bile acid concentrations (ng/mg) and percentages as well as Bai-gene abundance (log DNA) of dogs fed extruded, fresh, and raw foods

Item	EXT^*^	FRSH	FRZN	HFD	FD	SEM^†^	*P*-value
Total bile acids	3925.49^c^	6418.48^bc^	11090.00^a^	6799.77^b^	8892.27^ab^	784.3	<0.0001
Total primary bile acids	503.01^b^	4387.47^a^	786.08^b^	375.11^b^	623.62^b^	584.3	<0.0001
CA^‡^	411.48^b^	3909.30^a^	638.07^b^	313.98^b^	548.36^b^	389.3	<0.0001
CDCA	91.53^bc^	478.18^a^	148.01^b^	61.13^c^	75.26^bc^	42.65	<0.0001
Total secondary bile	3422.48^c^	2031.01^c^	10304.00^a^	6424.66^b^	8268.65^ab^	711.1	<0.0001
LCA	384.95^x^	177.05^x^	1857.72^z^	967.84^y^	714.74^y^	121.0	<0.0001
DCA	2920.31^cd^	1684.42^d^	8313.58^a^	5344.52^bc^	7500.68^ab^	662.2	<0.0001
UDCA	117.21^a^	169.54^a^	132.73^a^	112.30^ab^	53.23^b^	25.40	0.0006
Bile acids (% of total)							
Primary bile acids	12.38^y^	72.22^z^	6.70^y^	6.07^y^	6.88^y^	4.312	0.0001
CA	10.05^y^	64.18^z^	5.40^y^	5.15^y^	6.01^y^	3.960	0.0001
CDCA	2.33^y^	8.04^z^	1.30^xy^	0.92^x^	0.86^x^	0.5284	<0.0001
Secondary bile acids	87.62^z^	27.78^y^	93.30^z^	93.93^z^	93.12^z^	4.312	0.0001
LCA	10.33^bc^	2.35^d^	17.11^a^	14.71^ab^	8.47^c^	1.433	<0.0001
DCA	73.97^y^	22.62^x^	74.99^y^	77.54^yz^	83.99^z^	4.185	<0.0001
UDCA	3.33^a^	2.81^ab^	1.20^cd^	1.67^bc^	0.67^d^	0.3677	<0.0001
Bai-gene	5.14^a^	3.69^b^	5.48^a^	5.15^a^	3.93^b^	0.2616	<0.0001

^*^EXT: Chicken and Barley Recipe (Hill’s Pet Nutrition, Topeka, KS); FRSH: Chicken and White Rice Recipe (Just Food for Dogs, Irvine, CA); FRZN: Chicken Formula (Primal Pet Group, Fairfield, CA); HFD: Chicken and Sorghum Hybrid Freeze-Dried Formula (Primal Pet Group, Fairfield, CA); FD: Chicken Dinner Patties (Stella & Chewy’s, Milwaukee, WI).

^†^Pooled standard error of the mean.

^‡^CA, cholic acid; CDCA, chenodeoxycholic acid; LCA, lithocholic acid; DCA, deoxycholic acid; UDCA, ursodeoxycholic acid.

^a-d^Means lacking a common superscript differ (*P* < 0.05).

^x-z^Means lacking a common superscript differ using Wilcoxon (*P* < 0.05).

Dogs fed the FRZN diet had higher (*P* < 0.0001) fecal total fatty acid concentrations than dogs fed all other diets (**Table 5**). Also, dogs fed the EXT diet had higher (*P* < 0.0001) fecal total fecal fatty acid concentrations than dogs fed the FRSH diet. Many differences were noted in individual fatty acid concentrations, with oleate, palmitate, stearate, and linoleate being the most predominant in all groups. Dogs fed the FRZN diet had higher (*P* < 0.0001) fecal total sterol concentrations than dogs fed the FRSH, HFD, and FD diets. Also, dogs fed the EXT, HFD, and FD diets had higher (*P* < 0.0001) fecal total sterol concentrations than dogs fed the FRSH diet. Cholesterol was a predominant sterol in all treatment groups, with the highest (*P* < 0.0001) concentrations being present in the feces of dogs fed diets having the highest protein concentrations (FRZN; FD). Concentrations of β-sitosterol and sitostanol, plant sterols, were highest (*P* < 0.0001) in the feces of dogs fed the EXT diet.

**Table 5.** Fecal fatty acid and sterol concentrations (µg/mg) of dogs fed extruded, fresh, and raw foods

Item	EXT^*^	FRSH	FRZN	HFD	FD	SEM^†^	*P*-value
Total fatty acids	22.37^b^	17.92^c^	35.33^a^	18.64^bc^	20.99^bc^	2.837	<0.0001
α-linolenate	1.25^a^	0.14^b^	0.12^b^	0.15^b^	0.10^b^	0.0745	<0.0001
Arachidonate	1.45^b^	2.80^a^	2.12^ab^	1.92^ab^	1.89^ab^	0.2961	0.0089
cis-vaccenate	1.64	1.40	1.34	1.68	1.53	0.3562	0.3217
Docosanoate	0.58^a^	0.32^b^	0.15^c^	0.50^a^	0.47^a^	0.0892	<0.0001
Erucate	0.04	0.04	0.04	0.05	0.03	0.0061	0.0732
Gondoate	0.52^ab^	0.30^c^	0.61^a^	0.50^ab^	0.35^bc^	0.0768	<0.0001
Linoleate	4.48^a^	1.71^d^	2.95^bc^	2.29^c^	3.96^ab^	0.2010	<0.0001
Myristate	0.37	0.54	0.35	0.34	0.38	0.0717	0.6889
Nervonate	0.39^c^	0.49^bc^	0.87^a^	0.67^ab^	0.62^abc^	0.0802	0.0002
Oleate	4.23^b^	2.77^c^	15.30^a^	3.67^b^	3.99^b^	0.5248	<0.0001
Palmitate^‡^	4.52	4.26	5.54	3.44	4.13	0.9147	0.0086
Stearate^‡^	2.91	3.15	4.62	3.43	3.53	0.6419	0.0202
Total sterols	14.53^ab^	6.28^c^	16.98^a^	11.78^b^	12.89^b^	1.067	<0.0001
β-sitosterol	5.14^a^	0.74^c^	1.49^b^	1.75^b^	0.19^d^	0.1179	<0.0001
Brassicasterol	0.03^a^	0.01^c^	0.01^c^	0.02^b^	0.02^b^	0.0010	<0.0001
Campesterol	1.40^a^	0.25^c^	0.48^b^	0.62^b^	0.23^c^	0.0450	<0.0001
Cholestanol	0.16^c^	0.26^c^	0.69^a^	0.50^b^	0.57^b^	0.0322	<0.0001
Cholesterol	3.76^c^	4.70^bc^	13.79^a^	7.95^b^	11.55^a^	0.9682	<0.0001
Coprostanol	0.01^bc^	0.01^abc^	0.02^a^	0.02^ab^	0.01^c^	0.0011	0.0002
Fusosterol	0.29^a^	0.03^e^	0.06^d^	0.10^c^	0.14^b^	0.0077	<0.0001
Lathosterol	0.02^b^	0.03^b^	0.08^a^	0.05^a^	0.06^a^	0.0217	<0.0001
Sitostanol	2.92^a^	0.05^c^	0.06^c^	0.51^b^	0.05^c^	0.0631	<0.0001
Stigmasterol	0.79^a^	0.18^c^	0.29^b^	0.27^b^	0.07^d^	0.0178	<0.0001

^*^EXT: Chicken and Barley Recipe (Hill’s Pet Nutrition, Topeka, KS); FRSH: Chicken and White Rice Recipe (Just Food for Dogs, Irvine, CA); FRZN: Chicken Formula (Primal Pet Group, Fairfield, CA); HFD: Chicken and Sorghum Hybrid Freeze-Dried Formula (Primal Pet Group, Fairfield, CA); FD: Chicken Dinner Patties (Stella & Chewy’s, Milwaukee, WI).

^†^Pooled standard error of the mean.

^‡^Significant differences among treatments, but not after correction for multiple comparisons.

^a-e^Means lacking a common superscript differ (*P* < 0.05).

### *Fecal* Microbiota

Dogs fed all diets had a dysbiosis index in the normal range (**Table 6**). However, dogs fed the EXT diet had a lower (*P* = 0.0091) dysbiosis index than those fed the HFD diet. While differences were noted in the fecal abundances of bacterial taxa using qPCR, most were within the reference ranges (AlShawaqfeh et al., 2017). Two exceptions were the level of *Blautia*, which was lower than the reference range in dogs fed the FRSH diet and the level of *Turicibacter* that was higher than the reference range in dogs fed the EXT diet.

**Table 6.** Dysbiosis index and fecal bacterial abundance (log DNA/g feces) of dogs fed extruded, fresh, and raw foods*

Item	Reference range^†^	EXT^‡^	FRSH	FRZN	HFD	FD	SEM^‖^	*P*-value
Dysbiosis index	<0	−5.75^b^	−3.51^ab^	−3.87^ab^	−2.56^a^	−4.45^ab^	0.5998	0.0091
Total bacteria	—	11.15^ab^	11.24^a^	10.82^c^	11.13^ab^	11.03^b^	0.0637	<0.0001
*Faecalibacterium*	3.4 to 8.0	6.31^a^	5.63^b^	5.35^b^	5.71^ab^	4.26^c^	0.1784	<0.0001
*Turicibacter*	4.6 to 8.1	8.55^a^	7.12^bc^	6.88^c^	7.79^ab^	5.66^d^	0.2433	<0.0001
*Blautia*	9.5 to 11.0	9.95^yz^	8.69^x^	9.81^y^	10.12^yz^	10.15^z^	0.1239	<0.0001
*Fusobacterium*	7.0 to 10.3	8.64^bc^	8.45^c^	9.60^a^	9.08^b^	9.87^a^	0.1204	<0.0001
*Clostridium hiranonis*	5.1 to 7.1	6.13^xyz^	5.55^x^	6.50^z^	6.40^yz^	5.52^xy^	0.1958	0.0001
*Streptococcus*	1.9 to 8.0	3.80^b^	3.62^b^	4.01^b^	6.29^a^	2.85^b^	0.3988	<0.0001
*Escherichia coli*	0.9 to 8.0	3.72^c^	5.40^ab^	6.39^a^	4.66^bc^	5.94^ab^	0.3915	<0.0001
*Ruminococcus gnavus*	—	10.98^a^	10.12^b^	10.45^ab^	11.02^a^	9.44^c^	0.1592	<0.0001

*All treatment groups were negative for *Clostridium difficile.*

^†^ AlShawaqfeh et al., 2017.

^‡^EXT: Chicken and Barley Recipe (Hill’s Pet Nutrition, Topeka, KS); FRSH: Chicken and White Rice Recipe (Just Food for Dogs, Irvine, CA); FRZN: Chicken Formula (Primal Pet Group, Fairfield, CA); HFD: Chicken and Sorghum Hybrid Freeze-Dried Formula (Primal Pet Group, Fairfield, CA); FD: Chicken Dinner Patties (Stella & Chewy’s, Milwaukee, WI).

^‖^Pooled standard error of the mean.

^a-d^Means lacking a common superscript differ (*P* < 0.05).

^x-z^Means lacking a common superscript differ using Wilcoxon (*P* < 0.05).

**Figure 2.**
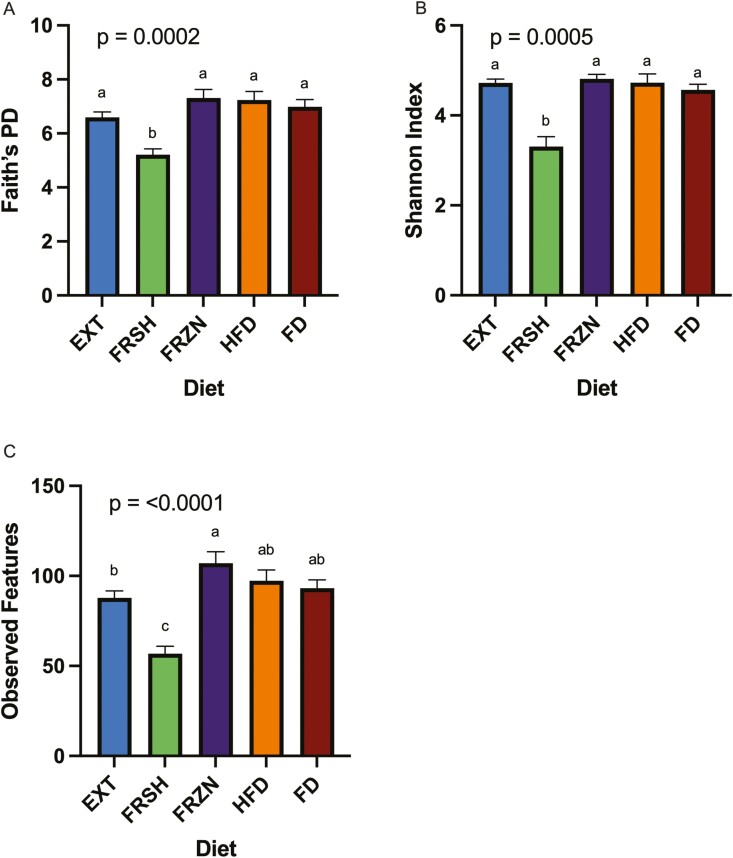
Alpha diversity plots of dogs fed extruded, fresh, and raw foods. A: Faith’s PD; B: Shannon Index; C: Observed Features. ^a-c^Means lacking a common superscript differ (*P* < 0.05).

**Figure 3.**
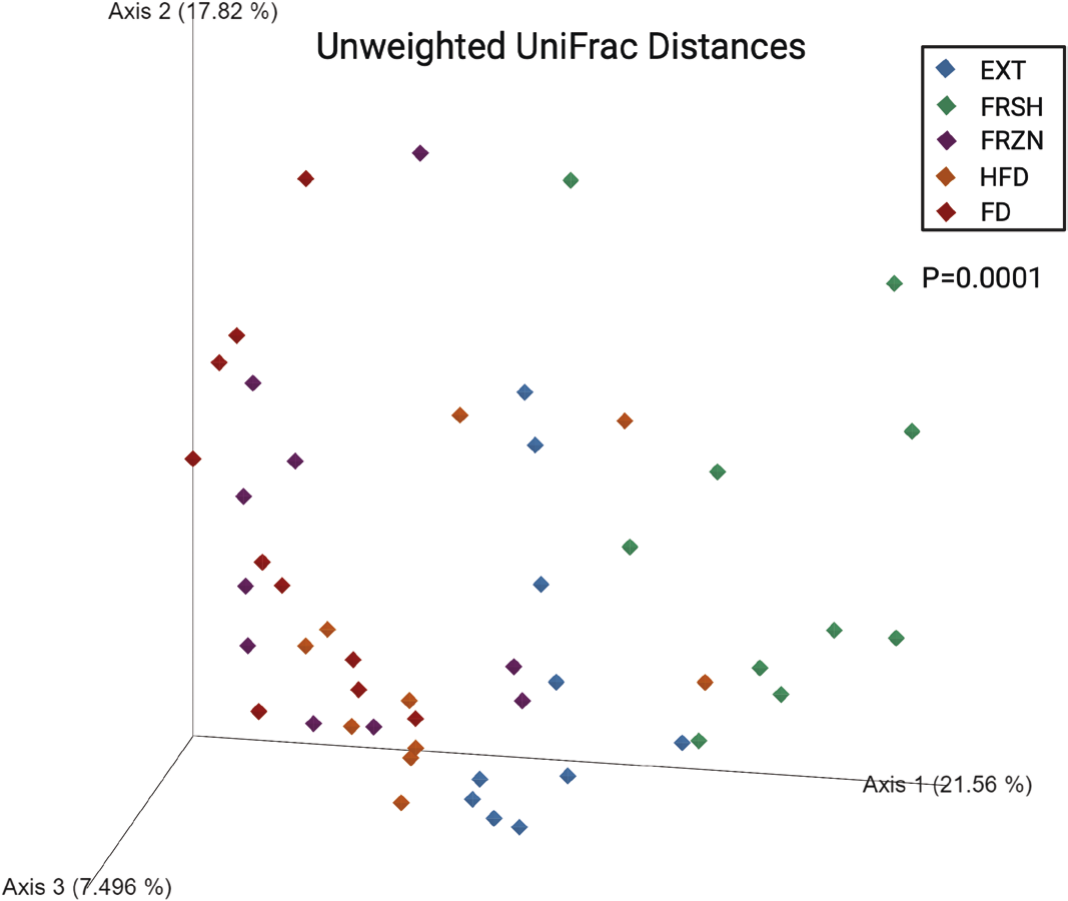
Beta diversity plot of unweighted UniFrac distances of dogs fed extruded, fresh, and raw foods.

**Figure 4.**
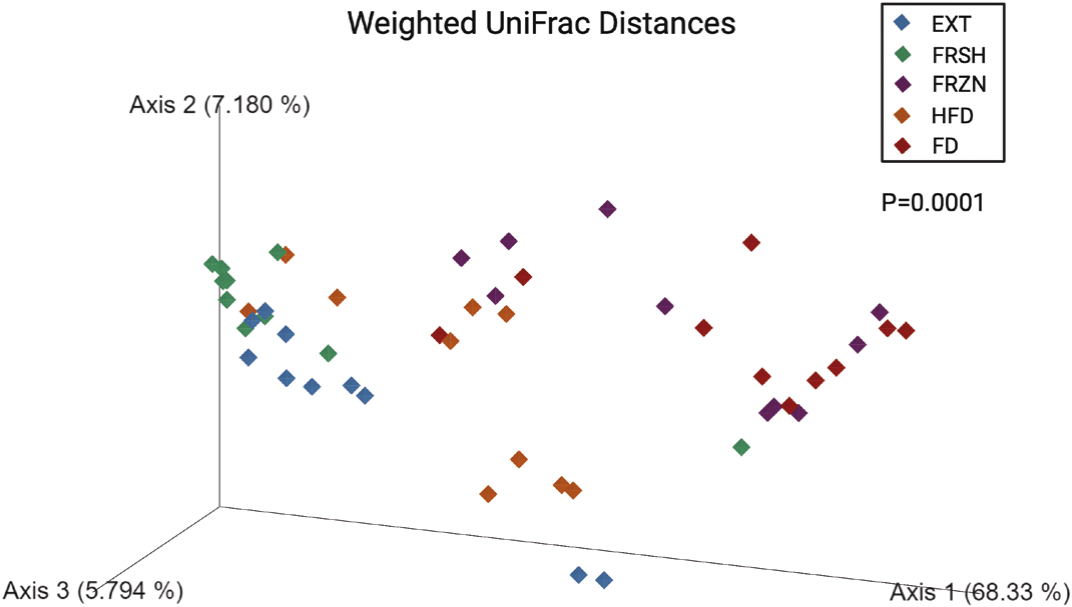
Beta diversity plot of weighted UniFrac distances of dogs fed extruded, fresh, and raw foods.

Using 16S rRNA gene amplicon sequencing, the fecal microbiota populations among dogs fed the treatment diets differed drastically. Bacterial alpha diversity indices (Faith’s PD, Shannon Index, and observed features) showed differences among treatments (**Figure 2**). Dogs fed the FRSH diet had a lower Faith’s PD (*P* = 0.0002), Shannon Index (*P* = 0.0005), and observed features (*P* < 0.0001) than dogs fed all other diets. Also, dogs fed the EXT diet had lower (*P* < 0.0001) observed features than dogs fed the FRZN diet. Beta diversity indices based on unweighted (**Figure 3**) and weighted (**Figure 4**) UniFrac distances showed large differences among treatments (*P* = 0.0001), with dogs fed the same diet tending to cluster together. Four fecal bacterial phyla and over 40 fecal bacterial genera were affected (*P* < 0.05) and 1 fecal phyla and 2 fecal genera tended to be affected (*P* ≤ 0.10) by dietary treatment (**Table 7**). Fecal Actinobacteria relative abundance was higher (*P* < 0.0001) in dogs fed the EXT or FRSH diets than those fed the FRZN, HFD, and FD diets. This difference was largely driven by the relative abundance of *Bifidobacterium*, which had the same pattern among treatments. Fecal Bacteroidota relative abundance was higher (*P* = 0.0005) in dogs fed the EXT, FRZN, HFD, and FD diets than those fed the FRSH diet. This difference was largely driven by the relative abundance of *Bacteroides*, which had the same pattern among treatments. Fecal Firmicutes’ relative abundance was higher (*P* < 0.0001) in dogs fed the EXT, FRSH, or HFD diets than those fed the FRZN or FD diets. Much of this difference was related to the difference in *Allobaculum* relative abundance, which had a similar pattern, as well as differences in other predominant genera (*Blautia*; *Dubosiella*; *Faecalibaculum*; *Lactobacillus*; *Peptoclostridium*; *Ruminococcus gnavus group*; *Turicibacter*; unclassified Erysipelotrichaceae) in Firmicutes. Fecal Fusobacteriota relative abundance was higher (*P* < 0.0001) in dogs fed the FRZN or FD diets than those fed the EXT, FRSH, or HFD diets. This was completely due to differences in *Fusobacterium* relative abundance, which had the same pattern.

**Table 7.** Predominant fecal bacterial phyla and genera (% of sequences) of dogs fed extruded, fresh, and raw foods

Phyla	Genera	EXT^*^	FRSH	FRZN	HFD	FD	SEM^†^	*P*-value
Actinobacteriota		14.53^a^	13.64^a^	4.81^b^	7.09^b^	4.67^b^	1.704	<0.0001
	*Adlercreutzia*	0.12	0.08	0.06	0.06	0.04	0.0221	0.6091
	*Bifidobacterium*	11.06^a^	11.79^a^	0.44^b^	4.40^b^	0.47^b^	1.473	<0.0001
	*Collinsella*	0.45^c^	0.41^d^	2.01^ab^	1.19^b^	2.98^a^	0.5706	<0.0001
	Coriobacteriaceae UCG-002	2.83^a^	1.32^b^	1.46^b^	1.31^b^	0.78^b^	0.6024	0.0009
	*Slackia*	0.03^b^	0.04^b^	0.78^a^	0.11^b^	0.38^ab^	0.1154	0.0038
Bacteroidota		8.37^a^	3.57^b^	10.03^a^	9.54^a^	11.91^a^	1.905	0.0005
	*Alloprevotella*	0.70^bc^	0.14^c^	1.78^abc^	2.85^ab^	3.87^a^	0.6238	0.0006
	*Bacteroides*	4.14^a^	2.53^b^	7.08^a^	4.70^a^	6.47^a^	1.343	0.0001
	Muribaculaceae	2.44^a^	0.85^ab^	0.56^b^	0.87^ab^	0.43^b^	0.4986	0.0022
	*Parabacteroides*	0.03	0.01	0.14	0.17	0.11	0.0364	0.1920
	*Prevotella*	1.03^a^	0.05^b^	0.06^b^	0.78^a^	0.03^b^	0.1724	<0.0001
	Prevotellaceae Ga6A1 group	0.02	0.00	0.38	0.14	0.91	0.3049	0.6900
	Rikenellaceae RC9 gut group	0.01^ab^	0.00^b^	0.02^a^	0.05^a^	0.10^a^	0.0143	0.0054
Firmicutes		72.22^a^	76.52^a^	51.48^b^	71.35^a^	44.66^b^	3.833	<0.0001
	*Allisonella*	0.12^ab^	0.02^bc^	0.00^c^	0.21^a^	0.02^b^	0.0405	0.0002
	*Allobaculum*	23.63^b^	57.73^a^	13.76^b^	23.78^b^	8.28^b^	5.565	<0.0001
	*Blautia*	3.81^ab^	0.42^c^	2.92^b^	3.78^ab^	5.08^a^	0.6419	<0.0001
	*Butyricicoccus*	0.18^a^	0.01^b^	0.18^a^	0.19^a^	0.08^ab^	0.0282	<0.0001
	*Catenibacterium*	0.42	0.01	0.01	0.03	0.01	0.1853	0.9302
	*Cellulosilyticum*	0.14^z^	0.00^y^	0.12^z^	0.00^y^	0.02^yz^	0.0362	0.0002
	*Clostridium sensu stricto 1*	0.34	0.60	1.82	1.06	1.16	0.4078	0.0543
	*Dubosiella*	3.59^a^	0.71^b^	0.96^ab^	2.42^ab^	1.62^ab^	0.8771	0.0156
	*Epulopiscium*	0.03	0.00	0.13	0.01	0.02	0.0313	0.1090
	*Erysipelatoclostridium*	0.24	0.00	0.07	0.00	0.03	0.0726	0.6886
	Erysipelotrichaceae UCG 003	0.38	0.00	0.00	0.03	0.00	0.1687	0.1727
	*Eubacterium*	0.09^b^	0.09^b^	0.10^b^	0.27^a^	0.16^ab^	0.0434	0.0001
	*Eubacterium brachy group*	0.00	0.00	0.00	0.11	0.00	0.0322	0.0858
	*Faecalibacterium*	1.26^a^	0.00^c^	0.79^ab^	0.51^ab^	0.25^b^	0.2154	<0.0001
	*Faecalibaculum*	4.86^a^	0.26^c^	0.28^c^	1.96^ab^	0.78^bc^	0.8463	<0.0001
	*Faecalitalea*	0.00^x^	0.00^x^	0.19^z^	0.00^xy^	0.09^yz^	0.0464	0.0001
	*Holdemanella*	1.03^a^	0.03^b^	0.03^b^	1.39^a^	0.47^ab^	0.3761	0.0002
	*Lachnoclostridium*	0.13^c^	0.30^abc^	0.43^ab^	0.21^bc^	0.61^a^	0.1576	0.0010
	*Lachnospira*	0.01^b^	0.03^b^	0.41^a^	0.06^b^	0.03^b^	0.0818	0.0028
	Lachnospiraceae NK4A136 group	0.02^xy^	0.00^x^	0.10^yz^	0.15^z^	0.10^yz^	0.0335	0.0001
	*Lactobacillus*	1.86^b^	7.49^a^	0.05^b^	0.71^b^	0.30^b^	1.700	<0.0001
	*Megamonas* ^‡^	2.88	0.57	0.47	1.91	0.89	1.036	0.0471
	*Negativibacillus*	0.00^y^	0.00^y^	0.03^yz^	0.13^z^	0.19^z^	0.0409	<0.0001
	*Paeniclostridium*	0.00^y^	0.00^yz^	0.14^yz^	0.00^y^	0.25^z^	0.0539	0.0018
	*Peptoclostridium*	3.58^bc^	0.93^c^	7.93^a^	5.03^ab^	8.32^a^	1.298	<0.0001
	*Peptococcus*	0.32^bc^	0.07^c^	2.24^a^	0.60^b^	1.86^a^	0.3570	<0.0001
	*Peptostreptococcus*	0.00	0.60	1.57	1.93	2.94	0.7556	0.2912
	*Phascolarctobacterium*	0.39^a^	0.04^b^	0.24^ab^	0.46^a^	0.40^a^	0.0739	0.0020
	*Romboutsia*	1.20^a^	0.47^b^	1.55^a^	0.89^ab^	1.13^a^	0.2553	0.0028
	*Ruminococcus gnavus group*	2.03^ab^	1.31^b^	3.66^a^	3.38^ab^	2.88^ab^	0.9469	0.0171
	*Ruminococcus gauvreauii group*	0.22^b^	0.00^c^	0.14^bc^	0.57^a^	0.10^bc^	0.0488	<0.0001
	*Ruminococcus torques group*	0.91^a^	0.05^b^	0.86^a^	0.45^a^	0.85^a^	0.1618	<0.0001
	*Sellimonas*	0.13	0.01	0.24	0.22	0.43	0.0757	0.1907
	*Streptococcus* ^‡^	0.01	0.44	0.01	4.41	0.00	0.9393	0.0375
	*Terrisporobacter*	0.08	0.04	0.27	0.10	0.09	0.0504	0.2606
	*Turicibacter*	5.13^a^	0.99^b^	0.14^b^	1.18^b^	0.03^b^	0.4913	<0.0001
	*UCG 005*	0.01^wx^	0.00^w^	0.17^yz^	0.06^xy^	0.42^z^	0.0642	<0.0001
	Unclassified in family Butyricicoccaceae	0.00	0.01	0.01	0.01	0.10	0.0219	0.9507
	Unclassified in family Erysipelotrichaceae	11.15^a^	2.81^b^	5.14^ab^	11.13^a^	1.78^b^	1.705	0.0002
	Unclassified in family Lachnospiraceae	1.49^a^	0.17^b^	2.08^a^	1.01^a^	1.17^a^	0.2898	<0.0001
	Unclassified in family Lachnospiraceae	0.30^bc^	0.26^c^	1.75^a^	0.76^ab^	1.32^a^	0.2681	<0.0001
	Unclassified in family Ruminococcaceae	0.01^bc^	0.00^cc^	0.10^a^	0.07^ab^	0.07^a^	0.0143	<0.0001
	Unclassified in family Ruminococcaceae	0.09	0.00	0.03	0.05	0.06	0.0199	0.2522
	Unclassified in order Erysipelotrichales	0.00^y^	0.00^y^	0.04^z^	0.01^yz^	0.07^z^	0.0118	<0.0001
Fusobacteriota		2.61^b^	3.34^b^	29.12^a^	9.25^b^	34.69^a^	2.691	<0.0001
	*Cetobacterium*	0.00	0.57	1.44	0.09	2.84	1.039	0.6221
	*Fusobacterium*	2.61^b^	2.77^b^	27.68^a^	9.16^b^	31.84^a^	2.810	<0.0001
Proteobacteria		2.27	2.93	4.54	2.76	4.07	0.6923	0.1014
	*Anaerobiospirillum*	0.60	0.08	0.85	0.44	0.30	0.2778	0.3533
	*Escherichia Shigella*	0.01^b^	0.94^ab^	1.23^a^	0.09^ab^	0.98^ab^	0.3751	0.0212
	*Parasutterella*	1.39	1.47	2.04	1.55	2.06	0.6254	0.6036
	*Sutterella*	0.24	0.42	0.38	0.67	0.72	0.2708	0.1194

^*^EXT: Chicken and Barley Recipe (Hill’s Pet Nutrition, Topeka, KS); FRSH: Chicken and White Rice Recipe (Just Food for Dogs, Irvine, CA); FRZN: Chicken Formula (Primal Pet Group, Fairfield, CA); HFD: Chicken and Sorghum Hybrid Freeze-Dried Formula (Primal Pet Group, Fairfield, CA); FD: Chicken Dinner Patties (Stella & Chewy’s, Milwaukee, WI).

^†^Pooled standard error of the mean.

^‡^Significant differences among treatments, but not after correction for multiple comparisons.

^a-d^Means lacking a common superscript differ (*P* < 0.05).

^w-z^Means lacking a common superscript differ using Wilcoxon (*P* < 0.05).

### *Serum Metabolite* Concentrations *and Hematology*

Many serum metabolite concentrations were different among groups, but all fell within normal ranges for all diets, with the exception of globulin, which was below the range for all diets, and albumin:globulin ratio that was high for all diets (**Table 8**). Similarly, several hematology markers differed among groups, but none of them were out of range for all diets, with the exception of white blood cell counts that were below the reference range for dogs fed the FRSH (5.40 × 10^3^/μL), HFD (5.47 × 10^3^/μL), and FD (4.95 × 10^3^/μL) diets (**Table 9**). Because the majority of these markers were within reference ranges, we do not believe that treatment differences have physiological relevance.

**Table 8.** Serum metabolite concentrations of dogs fed extruded, fresh, and raw foods

Item	Reference range	EXT^*^	FRSH	FRZN	HFD	FD	SEM^†^	*P*-value
Creatinine, mg/dL	0.5 to 1.5	0.60	0.64	0.65	0.66	0.63	0.0310	0.2640
BUN^‡^, mg/dL	6 to 30	13.50^c^	10.60^d^	15.00^bc^	16.50^b^	20.30^a^	0.9202	<0.0001
Total protein, g/dL	5.1 to 7.0	5.54	5.60	5.66	5.62	5.64	0.0845	0.4469
Albumin, g/dL	2.5 to 3.8	3.05^b^	3.10^ab^	3.15^ab^	3.16^ab^	3.20^a^	0.0765	0.0112
Globulin, g/dL	2.7 to 4.4	2.49	2.50	2.51	2.46	2.44	0.0639	0.6154
Albumin/globulin ratio^‖^	0.6 to 1.1	1.23	1.24	1.27	1.31	1.31	0.0524	0.0388
Calcium, mg/dL	7.6 to 11.4	9.91	9.91	9.89	10.00	9.89	0.1067	0.7652
Phosphorus, mg/dL	2.7 to 5.2	3.75	3.79	3.31	3.82	3.40	0.1675	0.0617
Sodium, mmol/L	141 to 152	145.70^ab^	146.60^a^	146.50^ab^	145.40^b^	145.90^ab^	0.4040	0.0285
Potassium, mmol/L	3.9 to 5.5	4.46	4.31	4.34	4.44	4.38	0.0623	0.0913
Sodium/potassium ratio^‖^	28 to 36	32.70	34.00	34.00	32.80	33.40	0.5189	0.0360
Chloride, mmol/L	107 to 118	112.30^ab^	113.20^ab^	113.80^ab^	112.10^b^	114.00^a^	0.5722	0.0086
Glucose, mg/dL	68 to 126	85.70	83.20	82.60	84.60	86.40	2.267	0.2325
ALP^‡^, U/L	7 to 92	46.20^a^	31.30^b^	17.60^c^	20.90^c^	16.20^c^	4.087	<0.0001
CALP^‡^, U/L	0 to 40	4.00^a^	2.60^b^	1.40^b^	3.80^ab^	2.20^b^	2.089	0.0102
ALT^‡^, U/L	8 to 65	27.70	22.20	23.30	20.70	24.50	3.180	0.8108
GGT^‡^, U/L	0 to 7	3.70^a^	3.50^ab^	3.00^ab^	2.90^b^	3.00^ab^	0.3029	0.0182
Total bilirubin, mg/dL	0.1 to 0.3	0.23^ab^	0.16^bc^	0.16^bc^	0.28^a^	0.15^c^	0.0297	0.0003
Creatine kinase, U/L	26 to 310	86.60^b^	98.40^b^	108.70^ab^	122.30^a^	105.50^ab^	7.912	0.0022
Cholesterol, mg/dL	129 to 297	189.90^b^	158.60^c^	214.80^a^	218.30^a^	226.40^a^	8.967	<0.0001
Triglycerides, mg/dL	32 to 154	58.00^a^	55.50^ab^	41.50^c^	45.50^bc^	43.50^c^	3.301	0.0001
Bicarbonate, mmol/L	16 to 24	22.50	22.40	22.00	22.80	21.40	0.6255	0.2827
Anion Gap	8 to 25	15.40	15.30	15.20	14.90	14.80	0.4438	0.7199
cPLI^‡^, µg/L	0 to 200	79.50^a^	57.60^ab^	62.70^ab^	57.20^ab^	49.00^b^	15.27	0.0319

^*^EXT: Chicken and Barley Recipe (Hill’s Pet Nutrition, Topeka, KS); FRSH: Chicken and White Rice Recipe (Just Food for Dogs, Irvine, CA); FRZN: Chicken Formula (Primal Pet Group, Fairfield, CA); HFD: Chicken and Sorghum Hybrid Freeze-Dried Formula (Primal Pet Group, Fairfield, CA); FD: Chicken Dinner Patties (Stella & Chewy’s, Milwaukee, WI).

^†^Pooled standard error of the mean.

^‡^BUN, blood urea nitrogen; ALP, alkaline phosphatase; CALP, corticosteroid-induced alkaline phosphatase; ALT, alanine aminotransferase; GGT, gamma-glutamyl transferase; cPLI, canine pancreatic lipase immunoreactivity.

^‖^Significant differences among treatments, but not after correction for multiple comparisons.

^a-d^Means lacking a common superscript differ (*P* < 0.05).

**Table 9.** Hematology of dogs fed extruded, fresh, and raw foods

Item	Reference range	EXT^*^	FRSH	FRZN	HFD	FD	SEM^†^	*P*-value
RBC^‡^, 10^6^/μL	5.50 to 8.50	6.98^b^	7.26^ab^	7.60^a^	7.26^ab^	7.24^ab^	0.1546	0.0202
Hemoglobin, g/dL	12.0 to 18.0	15.20^b^	15.76^ab^	16.67^a^	15.95^ab^	15.95^ab^	0.3875	0.0153
Hematocrit, %	35.0 to 52.0	45.93^b^	47.21^ab^	49.51^a^	47.57^ab^	47.65^ab^	1.099	0.0386
Mean cell volume, fl	58.0 to 76.0	65.79^a^	65.08^b^	65.04^b^	65.51^ab^	65.80^a^	0.4778	0.0013
MCH^‡^, pg	20.0 to 25.0	21.76^bc^	21.70^c^	21.90^abc^	21.96^ab^	22.02^a^	0.1762	0.0049
MCHC^‡^, g/dL	33.0 to 38.6	33.09^b^	33.38^ab^	33.66^a^	33.54^a^	33.46^ab^	0.1313	0.0027
Platelets, 10^3^/μL	200 to 700	266.70	263.20	306.70	296.30	296.60	32.14	0.0823
Mean platelet volume, fl	N/A	10.89^a^	10.52^b^	9.99^d^	10.45^bc^	10.23^cd^	0.1958	<0.0001
WBC^‡^, 10^3^/μL	6.00 to 17.00	6.33^a^	5.40^ab^	7.12^a^	5.47^ab^	4.95^b^	0.6303	0.0022
Neutrophil, 10^3^/μL	3.00 to 11.50	4.84	3.75	5.95	3.43	3.50	0.7991	0.0843
Lymphocyte, 10^3^/μL	1.00 to 4.80	1.58	1.59	1.61	1.53	1.32	0.1648	0.1585
Monocyte, 10^3^/μL	0.20 to 1.40	0.34	0.31	0.29	0.21	0.28	0.0363	0.0603
Eosinophil, 10^3^/μL	0.10 to 1.00	0.36^a^	0.12^b^	0.13^b^	0.12^b^	0.13^b^	0.0362	<0.0001
Basophil, 10^3^/μL	0.00 to 2.00	0.01	0.02	0.01	0.01	0.01	0.0034	0.5340
Neutrophil, %	N/A	66.00	66.17	68.93	63.17	66.94	2.382	0.4257
Lymphocyte, %	N/A	25.59	29.13	25.37	28.37	27.47	2.639	0.3678
Monocyte, %	N/A	5.22	5.75	4.38	4.22	5.60	0.5323	0.0814
Eosinophil, %	N/A	6.22^a^	2.09^b^	1.86^b^	2.22^b^	2.52^b^	0.7021	<0.0001
Basophil, %	N/A	0.18	0.30	0.13	0.14	0.22	0.0638	0.7227

^*^EXT: Chicken and Barley Recipe (Hill’s Pet Nutrition, Topeka, KS); FRSH: Chicken and White Rice Recipe (Just Food for Dogs, Irvine, CA); FRZN: Chicken Formula (Primal Pet Group, Fairfield, CA); HFD: Chicken and Sorghum Hybrid Freeze-Dried Formula (Primal Pet Group, Fairfield, CA); FD: Chicken Dinner Patties (Stella & Chewy’s, Milwaukee, WI).

^†^Pooled standard error of the mean.

^‡^RBC, red blood cells; MCH, mean corpuscular hemoglobin; MCHC, mean corpuscular hemoglobin concentration; WBC, white blood cells.

^a-d^Means lacking a common superscript differ (*P* < 0.05).

## Discussion

Due to the increasing number of owners choosing to feed alternative diet formats to their dogs, the current study aimed to expand the knowledge on fresh, frozen raw, and freeze-dried raw pet food formats by assessing total tract nutrient digestibility, and fecal characteristics, metabolites, and microbiota in healthy adult dogs (Geary et al., 2023). Not only were the processing formats dissimilar among the test diets but the ingredient profiles and macronutrient composition varied immensely. The main protein source for all diets was chicken, but EXT, FRSH, and HFD diets also contained grain-based ingredients, which will, to some extent, contribute to the total dietary protein. Notably, EXT was the only diet that contained chicken meal, an ingredient that undergoes high-heat processing during rendering.

The differences in BW among dietary treatments are likely explained by discrepancies between the metabolizable energy (ME) values listed on the dietary labels and the actual ME values. For example, the EXT and FD diets were labeled as containing 3,937 kcal ME/kg DM and 4,664 kcal ME/kg DM, respectively, but in both cases, these ME levels fell between estimates devised using modified Atwater and Atwater values. However, the nitrogen-corrected true ME (TME_n_) values measured in our previous study, but not available before this current dog study commenced, were higher for both diets (4,262 and 4,973 ME/kg DM, respectively; Geary et al., 2023); therefore, the dogs may have been overfed those diets. The ME on the label for HFD (4,602 kcal/kg DM) also fell between estimates using the Atwater and modified Atwater values, while the label on the FRZN diet (5,333 kcal/kg DM) exceeded both. Nevertheless, the TME_n_ for both HFD (5,401 kcal/kg DM) and FRZN (5,860 kcal/kg DM) were well over the label value (Geary et al., 2023), conceivably leading to moderate weight gain. On the other hand, the FRSH diet label estimate (4,971 kcal/kg DM) was above the modified Atwater, Atwater, and TME_n_ values (4,615 kcal/kg DM; Geary et al., 2023). Therefore, when dogs consumed the FRSH diet, they were likely underfed, resulting in weight loss. However, due to the weekly adjustments being made to diet intake, the weight fluctuations in this study were minor and did not influence the BCS. Ultimately, though, these deviations between bag labels and actual ME content emphasize the inappropriateness of the AAFCO requirement for the use of modified Atwater values to estimate ME content on labels, particularly for raw and fresh dog foods.

Dogs fed the raw diets in the current study had a much lower as-is fecal output (37.33 to 39.80 g/d) than reported elsewhere (101.6 g/d); however, the female beagle dogs in Algya et al. (2018) consumed approximately twice the DM intake for the raw diet as the FZN diet in the present study. The extruded diet in Algya et al. (2018) contained whole grain corn, meat and bone meal, and corn gluten meal as the top ingredients, two of which were present in the EXT diet in the present study, but further down the ingredient list. The raw diet in Algya et al. (2018) was also chicken-based but had the addition of sweet potatoes, which increased the nitrogen-free extract content compared to the raw diets in the present study. Fecal output in this study was similar to that of female beagle dogs fed a raw chicken diet reported by Beloshapka et al. (2012). Nevertheless, the as-is fecal output/DM intake was higher in both Algya et al. (2018) and Beloshapka et al. (2012) than the raw diets tested in this study. Unlike the present study, there were no differences in as-is or DM fecal output between the raw and extruded diet in Algya et al. (2018), perhaps due to differences in ingredient formulation and macronutrient concentrations. The as-is and DM fecal output of dogs fed the FRSH diet were similar to a previous study that studied the same diet (Do et al., 2021). As noted in the present study, the as-is and DM fecal output were lower in female beagle dogs fed the FRSH diets versus those fed an extruded diet (Do et al., 2021). Interestingly, the previous study reported a higher food intake than the present study, but the fecal output was lower than in the present study (Do et al., 2021), possibly due to differences in the animals studied or diet formulations. Furthermore, the digestibility of the FRSH diet in the present study was slightly lower than that reported by Do et al. (2021). In general, the digestibility of raw diets was higher in the present study than the raw chicken diet tested by Beloshapka et al. (2012). Several factors can impact a diet’s digestibility, including particle size, processing conditions, macronutrient and ingredient composition, animal age, and food intake (Khan et al., 2003). The ingredients and macronutrient compositions of the raw diets across studies were not comparable, demonstrating that fecal output and digestibility are likely related to other factors such the incorporated ingredients and individual dog differences (breed, age, sex, MER) in addition to the type and extent of ingredient/diet processing.

The differences observed in average fecal score between EXT and FRSH compared to FRZN and FD are likely due to the differences in digestibility and macronutrient composition. Although the FD diet’s average fecal score was normal (2 to 3), the fecal scores varied from being dry and ashy (1) to diarrhea (5). We hypothesized that diarrhea was primarily caused by its lack of fiber, as fiber is essential for stool quality (Lappin et al., 2021). The raw diets tested in this study had lower fecal scores than the EXT diet, as reported in other studies (Beloshapka et al., 2012; Freeman et al., 2013; Algya et al., 2018; Hiney et al., 2021). Fecal pH was relatively high in the raw diets tested, as reported in a prior study (Beloshapka et al., 2012), likely due to containing low amounts of fiber and carbohydrates and high quantities of protein. As demonstrated by Beloshapka et al. (2012), fecal pH was associated with SCFA concentrations, with dogs fed raw diets having low fecal SCFA concentrations. Sandri et al. (2019) reported no difference in SCFA concentrations between border collie dogs fed beef raw meat with cooked carbohydrates and beef extruded diets; however, the raw diets in that study were formulated to contain much more fermentable material in the form of cooked pulses. BCFA were likely highest in dogs fed the HFD diet due to lower protein and amino acid (Geary et al., 2023) digestibility. The fecal pH of dogs fed the FRSH diet in the present study was lower than dogs fed the same diet by Do et al. (2021), but comparable to that tested by Geary et al. (2022). However, it is unclear why dogs fed the FRSH diet had a low fecal pH and high butyrate concentrations because the fiber and resistant starch content of this diet was not substantial.

Total and secondary bile acids were highest in dogs fed the FRZN and FD diets, possibly due to their high fat and low fiber inclusion levels (Reddy et al., 1980, 1988). Interesting differences in the primary and secondary bile acids concentrations and ratios were observed among diets. Primary bile acids, cholic acid (CA), and chenodeoxycholic acid (CDCA) are synthesized from cholesterol in the liver (Wahlström et al., 2016). Although an enzyme in the liver does most of the bile acid conjugation with either taurine or glycine, several bacterial species can conjugate bile acids and even more species can deconjugate them (Guzior and Quinn, 2021). Most bile acids (approximately 95%) are reabsorbed in the ileum and recycled to the liver (Borgström et al., 1968), but the remaining fraction of the primary bile acids are converted to secondary bile acids, deoxycholic acid (DCA) and lithocholic acid (LCA), in the colon by a select few bacteria, such as *Clostridium hiranonis* (Wahlström et al., 2016). The bai gene is conserved in bacteria that convert primary to secondary bile acids via 7α-dehydroxylation (Funabashi et al., 2020). In a healthy condition, dogs typically have a higher ratio of secondary to primary fecal bile acids, which is reversed or altered in a state of dysbiosis, such as in disease (Blake et al., 2019; Galler et al., 2022) or antibiotic administration (Belchik et al., 2023). It has also been shown that greater primary bile acids are associated with secretory diarrhea (Suchodolski et al., 2022). A reduction in primary to secondary bile acid conversion is usually accompanied by a decrease in *C. hiranonis* (Galler et al., 2022; Belchik et al., 2023). Interestingly, dogs fed the FRSH diet in the current study had a very low secondary: primary bile acid ratio, but the exact reason for it is unclear. First, although the fecal abundance of *C. hiranonis* in dogs fed the FRSH (5.55 log DNA/g feces) and FD (5.52 log DNA/g feces) diets were significantly lower than in dogs fed the FRZN (6.50 log DNA/g feces) diet, the abundance was within the normal range (5.1 to 7.1 log DNA/g feces). Second, while the bai gene abundance, which encodes for the enzyme that converts primary to secondary bile acid, was low in dogs fed the FRSH diet, it was also low in dogs fed the FD diet. Third, the low fecal pH of dogs fed the FRSH diet may have contributed to lower primary to secondary bile acid conversion (Bingham, 2000); however, fecal pH was not statistically different from that of dogs fed the EXT diet but may have physiological ramifications by inactivating the enzyme to a greater extent. Fourthly, although conjugated and unconjugated bile acids were not assessed in this study, it has been shown that a decrease in pH can cause unconjugated bile acids to precipitate, which causes there to be more insoluble bile acids, therefore decreasing the conversion from primary to secondary bile acids (Fini and Roda, 1987). Because we measured the abundance of the bai gene and *C. hiranonis* and not the activity of the enzyme, the abundance of *C. hiranonis* could be within normal ranges, but the activity of the enzyme responsible for conversion could be inhibited by the low pH caused by the FRSH diet. It appears that specific ingredients or macronutrient concentrations may impact bile acid concentrations more than the processing format, but more research is needed for confirmation.

Fecal fatty acids and plant sterols present in feces primarily indicate dietary indigestion or malabsorption because they are unable to be synthesized by dogs in the gastrointestinal tract (Montoro-Huguet et al., 2021). Sterols may also be metabolized by bacteria in the colon. It has been proposed that *Faecalibacterium* could be involved in plant sterol metabolism and that sterols may influence *Faecalibacterium* abundance (Galler et al., 2022). More research into the functions of docosanoic (behenic) acid, gondoic acid, linoleic acid, oleic acid, and stearic acid in the gastrointestinal tract is necessary to determine the causes for and implications of their fluctuations in the feces. In regard to sterols, β-sitosterol, brassicasterol, campesterol, fusosterol, sitostanol, and stigmasterol were highest in dogs fed the EXT diet, possibly due to the higher inclusion of plant ingredients, such as grains. Fecal cholesterol was much higher in dogs fed the raw diets, agreeing with data from a prior study (Schmidt et al., 2018), perhaps because of the high animal content of these diets. Cholestenol can be converted to cholesterol in mammals (Frantz et al., 1959) and lathosterol is an intermediate of cholesterol synthesis. In humans, much of the unabsorbed cholesterol is converted to coprostanol by bacteria (Veiga et al., 2005), but in dogs, the conversion to coprostanol is much lower (Leeming et al., 1996). The research on fecal sterols is scarce, making it more challenging to speculate on why the fecal concentrations of the sterols shifted due to the consumption of different diets.

Although the dysbiosis index is generally used for diseased populations and this population is only comprised of healthy dogs, we ran the dysbiosis index after observing the inverted bile acid ratio for the FRSH diet. All diets had negative dysbiosis indices, indicating that all animals had healthy fecal microbiomes throughout the study, even though dogs fed the FRSH diet had a low bile acid ratio and dogs fed the FD diet had intermittent diarrhea. Similar to Schmidt et al. (2018), fecal *E.coli* had a higher abundance, and fecal *Faecalibacterium* had a lower abundance in dogs fed the raw diets (FRZN and FD) than those fed the EXT diet. Although there was no change in the dysbiosis index, the fecal microbiota populations shifted drastically among diets.

The diets differed substantially in quantities of fiber, types of fiber, and nutrient digestibility. All of these factors are known to impact what substrates are available for microbial fermentation in the colon. Feed intake also impacts substrate availability. Due to dogs being underfed on the FRSH diet, there was less substrate available for fermentation. Likewise, dogs being overfed on the FRZN, HFD, and FD diets likely caused there to be an excess of substrate reaching the colon. With the exception of the EXT diet, all diets had very little fiber. The fiber content of the FRSH diet tested in the current study was much lower (2.21% DM) than that tested in other studies (7.09% DM; 11.42% DM; Do et al., 2021; Geary et al., 2022). The FRZN and HFD diets tested in the current study also had slightly lower fiber concentrations (3.48% and 3.88% DM) than those tested in a previous study (5.62% and 5.71% DM; Oba et al., 2023).

Dogs fed raw diets in the present study had similar relative abundances of fecal Actinobacteria, Fusobacteria, and Proteobacteria as dogs in a previous study (Algya et al., 2018). However, dogs fed raw diets in the current study had much lower fecal Bacteroidetes relative abundance and much higher fecal Firmicutes relative abundance than dogs fed raw diets in the study by Algya et al. (2018). As reported in previous studies (Kim et al., 2017; Sandri et al., 2017; Algya et al., 2018; Schmidt et al., 2018; Xu et al., 2021), the relative abundance of fecal Fusobacteria was higher in dogs fed raw diets (FRZN and FD) than those fed the EXT diet in the current study. Also, in the current study, the relative abundances of fecal Actinobacteria and Firmicutes were lower in dogs fed the raw diets than those fed the EXT diet, consistent with prior studies (Algya et al., 2018; Schmidt et al., 2018). This response was opposite to that reported by Sandri et al. (2017), however. There were no differences in relative abundances of fecal Bacteroidota and Proteobacteria between dogs fed the raw diets and EXT diet in the current study, agreeing with that of Kim et al. (2017) and Sandri et al. (2017), but not Algya et al. (2018), Schmidt et al. (2018), and Xu et al. (2021), which all reported a higher Proteobacteria relative abundance in dogs fed raw vs. extruded diets. Although Proteobacteria is well-known for containing the genera *Campylobacter, Escherichia coli,* and *Salmonella* that are common opportunistic pathogens, Proteobacteria play a role in normal gastrointestinal function, with their abundance varying widely among healthy dogs (Moon et al., 2018). Dogs fed raw diets in the current study also had a lower relative abundance of fecal Fusobacteria and a higher relative abundance of fecal Firmicutes than dogs studied by Beloshapka et al. (2013), but comparable relative abundances of fecal Bacteroidetes, Proteobacteria, and Actinobacteria. The relative abundances of fecal Actinobacteria, Firmicutes, and Fusobacteria were higher in dogs fed the FRSH diet in the current study than dogs fed the same diet in a previous study (Do et al., 2021). The relative abundances of fecal Bacteroidetes and Proteobacteria in dogs fed the FRSH diet of the current study were lower than those studied by Do et al. (2021).

In Algya et al. (2018), fecal *Bacteroides* and *Sutterella* relative abundances were elevated in dogs fed the raw diet than those fed the extruded diet, which was unlike that of the present study. Aligning with the results of the current study, relative abundances of fecal *Bifidobacterium* (Sandri et al., 2017; Schmidt et al., 2018) and *Turicibacter* (Sandri et al., 2017) were much lower in dogs fed the raw diet versus the EXT diet, and the relative abundance of *E. Shigella* (Sandri et al., 2017) was greater in the raw diet. *Turicibacter* is saccharolytic and has been shown to increase with the addition of fiber to the diet, which is perhaps why this genus was elevated in dogs fed the EXT diet (Jackson and Jewell, 2018). Again, consistent with the results of the present study, the relative abundance of fecal *Fusobacterium* was elevated in dogs fed a raw diet than those fed an extruded diet (Sandri et al., 2017; Algya et al., 2018; Schmidt et al., 2018). *Fusobacterium* uses amino acids for carbon and energy. Thus, it plays a vital role in the colonic fermentation of amino acids and protein metabolism (Potrykus et al., 2007). Therefore, its considerable elevation in dogs fed the two diets with the highest protein concentrations was not surprising. The relative abundances of fecal *Prevotella, Butyricicoccus,* and *Holdemanella* were greater in dogs fed the EXT diet than those fed the FRSH diet, aligning with a previous study’s results (Do et al., 2021). *Prevotella* was likely higher in the feces of dogs fed the EXT diet because it had a higher fiber and soluble carbohydrate content (Tett et al., 2019). *Allobaculum,* a butyrate producer, was higher in dogs fed the FRSH diet than in dogs fed all other diets, potentially contributing to the higher butyrate concentrations in dogs fed that diet (Greetham et al., 2004). The low fecal pH of dogs fed the FRSH diet might be responsible for the lowered alpha diversity and higher abundance of *Lactobacillus,* an acid-tolerant genus. The *Lactobacillus* genus is widely regarded as being beneficial, with various *Lactobacillus* strains being used as a probiotic; however, an extreme increase in *Lactobacillus* coupled with lower alpha diversity indicates a less diverse microbiome that may be more susceptible to disease (Suchodolski et al., 2022).

In conclusion, while all diets tested in the present study had high (>80%) apparent total tract macronutrient digestibilities, the EXT diet had lower digestibility than the other dietary formats. Consequently, fecal output was higher in dogs fed the EXT diet than those fed the other diet formats. Fecal pH was lower in dogs fed the EXT and FRSH diets than those fed the other diets. Mean fecal scores were lower in dogs fed FRZN and FD than those fed EXT and FRSH; however, there was a notable variability in the fecal scores of dogs fed the FD diet, with several scores falling outside the normal range. Dogs fed the EXT and FRSH diets had the highest fecal SCFA concentrations, with dogs fed the FRSH diet having the highest fecal butyrate concentrations. Dogs fed the HFD diet had the greatest fecal BCFA and indole concentrations. The fecal microbiota populations varied substantially among dogs fed the different diets, with beta diversity analyses showing that dogs tended to cluster according to diet. Although there were several differences observed among the diets tested in this study, the effects of ingredient composition, macronutrient profile, and processing conditions cannot be distinguished from one another. Future studies should be conducted to assess the effects due to processing, without differences in ingredient inclusion or macronutrient composition.
